# Supplementary material for: Quantifying the unknown impact of segmentation uncertainty on image-based simulations
Source: Nat Commun. 2021 Sep 14;12:5414. doi: 10.1038/s41467-021-25493-8 (PMC8440761; doi:10.1038/s41467-021-25493-8)
Supplement: Supplementary file 1 — Supplementary Information [file 41467_2021_25493_MOESM1_ESM.pdf]

Supplementary Information: Quantifying the unknown impact of segmentation uncertainty on image-based simulations

Michael C. Krygier[1], Tyler LaBonte[2], Carianne Martinez[2], Chance Norris[3], Krish Sharma[2], Lincoln N. Collins[1], Partha P. Mukherjee[3], and Scott A. Roberts*[1]

[1]Engineering Sciences Center, Sandia National Laboratories, Albuquerque, New Mexico, USA

[2]Applied Machine Intelligence and Application Engineering, Sandia National Laboratories, Albuquerque, New Mexico, USA

[3]School of Mechanical Engineering, Purdue University, West Lafayette, Indiana, USA

Multi-class EQUIPS Workflow

In the main manuscript, we primarily presented results for binarized images, where each voxel is classified into one of two phases. However, the EQUIPS workflow is equally applicable to images where there are multiple classes to segment, as we present in this supplementary information. We demonstrate this approach on a manufactured five-class image with a nominal segmentation shown in Supplementary Figure 1.

Consider a 3D image that is composed of a set of n_c segmented classes, $C = \{1, 2, \ldots, n_c\}$. The probability map for a multi-class image segmentation is generated identically to the binary case ($n_c = 2$). Namely,

$$\epsilon_{v,i} = \frac{1}{N} \sum_{k=1}^{N} p_{v,i}^{k}, \tag{S1}$$

where $\epsilon_{v,i}$ is the probability of voxel v being in class i, N is the number of image segmentation samples, and $p_{v,i}^{k}$ is the binarized value of voxel v being in class i in sample k. Here, $p \in \{0, 1\}$ is the binarized value inside voxel v, where $p = 1$ means that the voxel is in class i while $p = 0$ means that the voxel is not in class i.

The key to exploring segmentation uncertainty for multi-class problems, which is not obvious in the binary case, is that a separate probability map exists for each class i. As a result, the class probabilities in each voxel v must sum to unity:

$$\sum_{i \in C} \epsilon_{v,i} = 1. \tag{S2}$$

In the binary case ($n_c = 2$), adding Eq. (S1) together for each class $C = \{1, 2\}$ results in the simple identity $\epsilon_{v,1} = 1 - \epsilon_{v,2}$, as it must. For this reason, only one probability map is necessary to describe segmentation uncertainty in the binary case, as we have used throughout the main paper.

*Corresponding Author: sarober@sandia.gov

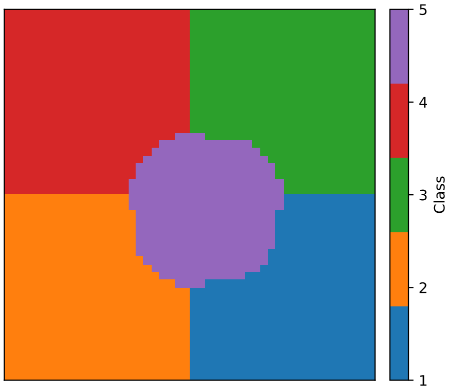

Supplementary Figure 1: **Nominal multi-class segmentation.** Illustration of a manufactured multi-class ($n_c = 5$) image segmentation, where each color represents a unique material class.

Let A represent a segmented multi-class image. Each voxel v in the segmented image has a unique class label such that $A_v \in C$, where A_v specifies a single voxel v in this image segmentation. In a two-class image, A is a *binarized* image segmentation. The nominal image segmentation of a multi-class image ($\mathcal{N}$, Supplementary Figure 1) is made by assigning each voxel to the class for which the probability is highest,

$$\mathcal{N}_v = \arg\max_{i \in C}(\epsilon_{v,i}), \tag{S3}$$

where $\mathcal{N}_v$ is simply the nominal segmentation for voxel v. The arg max operator returns the value of c when class c has the highest probability of all classes in C. In the binary image case, this is equivalent to defining $\mathcal{N}_v$ by $\epsilon_{v,1} > 0.5$ as was done throughout the main manuscript. It is also useful to specify the nominal segmentation *omitting* class $c \in C$ from the arg max operator:

$$\mathcal{N}_v^c = \arg\max_{i \in C,\, i \neq c}(\epsilon_{v,i}). \tag{S4}$$

We probe the uncertainty of this multi-class segmentation on a single class $c \in C$ at a time following Supplementary Algorithm 1. To generate a *percentile segmentation* where class c is above the percentile threshold α, first set all voxels where $\epsilon_{v,c} > \alpha$ to c. The remaining unassigned voxels are assigned to the most probable class other than c, $\mathcal{N}_v^c$ using Eq. (S4). The resulting image is the multi-class equivalent to the percentile segmentations of binary images in the main manuscript.

We demonstrate Supplementary Algorithm 1 by probing the probability maps of our manufactured multi-class image Supplementary Figure 1, with the results shown in Supplementary Figure 2. The per-class probability maps shown in the first row of images (Supplementary Figure 2(a-e)) were manufactured to generate the nominal segmentations shown in the second row (Supplementary Figure 2(f-j)). As in the main manuscript, we want to probe uncertainties for each class at multiple probability values, in this case 10%, 50%, and 90%, the results of which are shown in the remaining three rows (Supplementary Figure 2(k-y)).

As expected, for higher percentile values, the area of the probed class is smaller, indicating that fewer voxels have a high probability of being in that class. Whereas for lower percentile values, the area of that class is larger. For the high percentile values, the voxels no longer in class c are assigned to the most probable other class, which is most frequently (but not necessarily) the nearest neighboring class. The boundary between any two classes that are not c remains unchanged.

In the binary case, the nominal segmentation corresponds to the 50% probability values because of Eq. (S2). In contrast, selecting a percentile value of 50% from a multi-class probability map does

Supplementary Algorithm 1 Probe class c segmentation uncertainty

Require: Image I segmented into $C = \{1, 2, \ldots, n_c\}$ classes and represented by A, and percentile threshold value α.

Generate probability maps $\epsilon_{v,i}$ for voxel v and $i, c \in C$.
for all $v \in I$ **do**
    if $\epsilon_{v,c} > \alpha$ **then**
        $A_v = c$.
    else
        $A_v = \mathcal{N}_v^c$.
return A

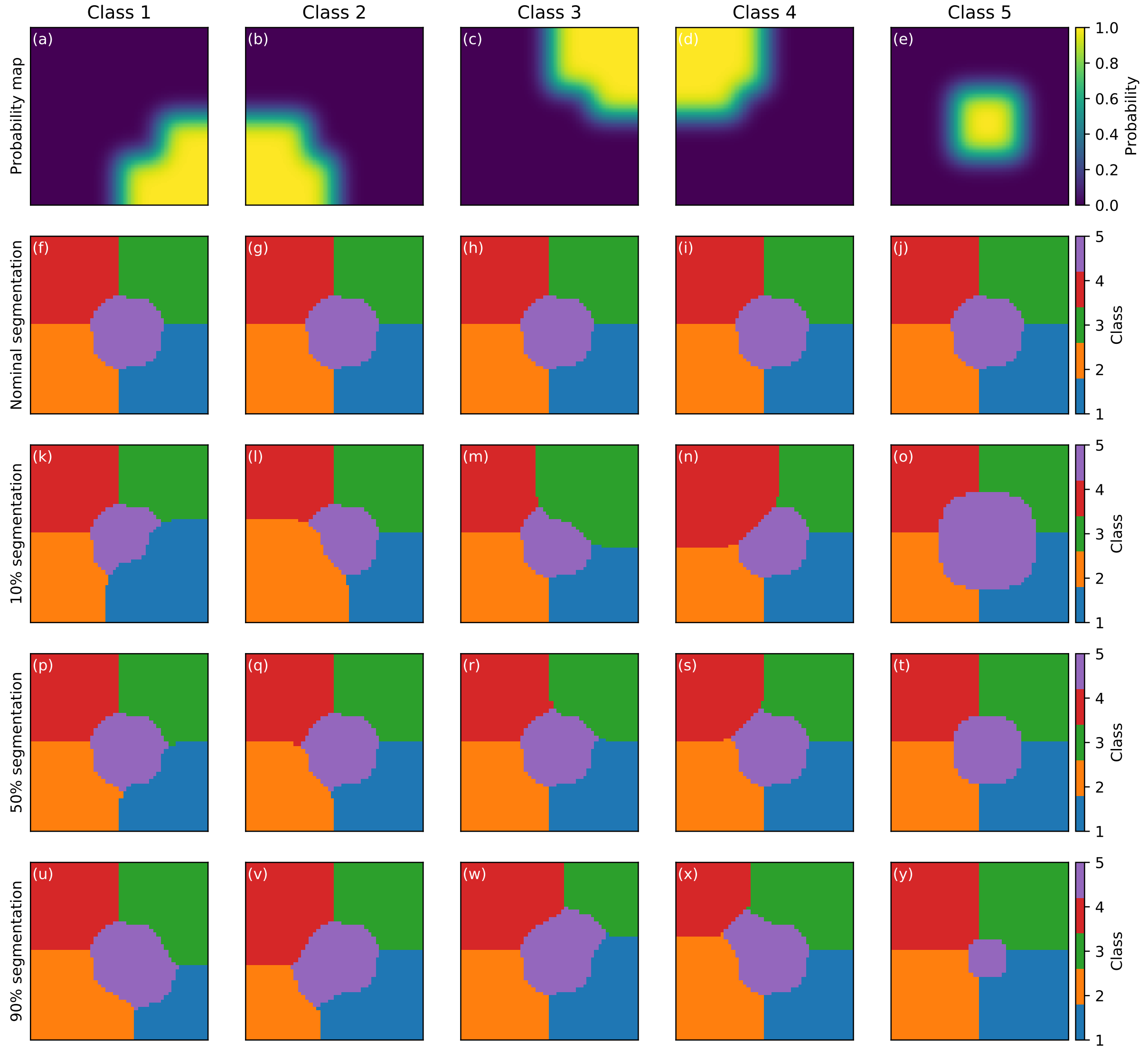

Supplementary Figure 2: **Effect of probing multi-class probability maps.** Each column represents each of the five classes in the multi-class image. The first row shows the probability map, ϵ_i, for each class i. The second row depicts the nominal class segmentations, $\mathcal{N}$. The third, fourth, and fifth rows illustrate the $(10, 50, 90)$-percentile segmentations for each class, respectively.

not return the nominal segmentation. The explanation for this observation is simple. When $n_c > 2$, the maximal probability value for any given voxel near the boundary between classes is most likely less than 0.5. Therefore, the 50-percentile segmentation sets a higher probability threshold than the nominal segmentation, resulting in a smaller area for class c than in the nominal segmentation.

The EQUIPS workflow allows for probing the segmentation uncertainty of one single class from a multi-class image at a time. However, one can imagine probing the segmentation uncertainty of multiple classes from a multi-class image simultaneously. An approach similar to the one proposed in this section can be envisioned. Doing this, however, would necessarily require a constraint on how the percentile values of the selected classes are chosen to satisfy Eq. (S2). Because there is some ambiguity to the authors on how to properly constrain percentile value selections, we omit further generalization of this approach.
